# Growth Stage-, Organ- and Time-Dependent Salt Tolerance of Halophyte *Tripolium pannonicum* (Jacq.) Dobrocz.

**DOI:** 10.3390/life13020462

**Published:** 2023-02-07

**Authors:** Agnieszka Ludwiczak, Anna Ciarkowska, Ahmad Rajabi Dehnavi, Stefany Cárdenas-Pérez, Agnieszka Piernik

**Affiliations:** 1 Department of Geobotany and Landscape Planning, Faculty of Biological and Veterinary Sciences, Nicolaus Copernicus University in Toruń, 87-100 Toruń, Poland; 2 Department of Biochemistry, Faculty of Biological and Veterinary Sciences, Nicolaus Copernicus University in Toruń, 87-100 Toruń, Poland; 3 Department of Agronomy and Plant Breeding, College of Agriculture, Isfahan University of Technology, Isfahan 84156-83111, Iran

**Keywords:** salinity, halophytes, *Tripolium pannonicum*, proline, hydrogen peroxide, antioxidant enzymes

## Abstract

*Tripolium pannonicum* (Jacq.) Dobrocz. is a member of the diverse group of halophytes with the potential for the desalination and reclamation of degraded land. The adaptive processes of *T. pannonicum* to salinity habitats are still not well recognized. Therefore, we evaluated the effect of NaCl (0, 200, 400, and 800 mM) on: (1) two plant growth stages, (2) the activity of antioxidant enzymes and concentration of H_2_O_2_ and the proline in roots, stems, and leaves, and (3) the effect of long- and short-term salt stress on physiological responses. Germination, pot experiments, and a biochemical analysis were performed. The effective *T. pannonicum*’s seed germination was achieved in the control. We demonstrated that halophyte’s organs do not simply tolerate high-salt conditions. The activities of APX, POD, and catalase observed at 400 mM and 800 mM NaCl were varied between organs and revealed the following pattern: root > leaves > stem. Proline was preferentially accumulated in leaves that were more salt-tolerant than other organs. Salt stress enhanced the activity of antioxidant enzymes and concentrations of salinity stress indicators in a time-dependent manner. Our study has indicated that salt tolerance is a complex mechanism that depends on the growth phase, organs, and duration of salinity exposure. The results have potential for further proteomic and metabolomic analyses of adaptive salt tolerance processes.

## 1. Introduction

Soil salinity is a dynamic and globally spreading issue for over one hundred countries [1]. Salinity affects almost all stages of plant development and causes osmotic stress and ionic and nutrient imbalance [1,2]. Halophytes are one of the most suitable models of salt stress tolerance mechanisms, due to their salt resistance [3]. The newest reports indicated that halophytes have considerable potential for the restoration of salt contaminated lands and potential for the phytosanitation and phytoremediation of the soil [4].

The adaptiveness of *T. pannonicum* to salinity habitats are poorly studied and ‘adaptive plant strategies’ are not explained. Therefore, the experimental model in our studies is the sea aster *Tripolium pannonicum* (Jacq.) Dobrocz. (formerly *Aster tripolium* L.) from the *Asteraceae* family, which grows in the salt marshes and coastal areas of temperate zones and in non-tidal saline areas [5]. The plant is also part of highly specialized habitats: Atlantic salt meadows (1330) and inland salt meadows (1340) listed on the Habitats Directive-Natura 2000 and the European Red List of Habitats [6]. As a plant from the coastal areas, *T. pannonicum* is periodically flooded with sea water while in inland habitats are surrounded by outflows of salty groundwater and are classified as coastal wetland-specific species [7]. This species tolerates long-term flooding, making it more successful than other halophytes that cannot survive long-term flooding [8]. *T. pannonicum* is present also in saline, moist, and nitrogen-rich anthropogenic habitats, e.g., the surroundings of the soda factory in Inowrocław (Poland) with the highest maximum salinity of 140 dS·m^−1^. However, in natural habitats, such as the nature reserve in Ciechocinek (Poland), salinity can be lower (1.58–38.5 S·m^−1^) [9]. Piernik identified that the ecological growth optima of *T. pannonicum* correspond with very strong saline soils [9]. The variability of the parental habitat’s salinity can be the basis of the extreme environmental adaptation of this plant. In addition, the ‘adaptive plant strategies’ may differ depending on the phase of growth, the plant’s organs, and the length of time of exposure to the stress factors and even between and within species [10]. Different halophytes can express different salt stress adaptation strategies that are essential both in the context of their protection/restoration and of the future saline agriculture development [11]. Enhancing ROS and/or osmolyte production and antioxidant defence mechanism improvement are the most documented examples [11,12]. Therefore, studies on the not well-examined endangered halophyte *T. pannonicum* can help to better understand the plant’s response to salinity stress and plant-environment relations, especially in the context of extreme climate change and habitat disturbances [13]. 

The initial growth, occurring at the germination and seedling stages, can influence a plant’s capacity to capture resources in later growth when competition for light and soil nutrients becomes more intense [14,15]. Therefore, successful germination and seedling development are crucial steps in the effective growth of a new plant. The salt tolerance of the germination differs between halophytes, therefore it is essential to evaluate the salinity effect on the not well-studied halophyte species. A common effect of abiotic and biotic stressors is an excessive production of the reactive oxygen species (ROS), such as hydrogen peroxide (H_2_O_2_) causing cellular oxidative stress and damage of the crucial macromolecules [3,16,17] and osmolytes (such as proline or glycine betaine) [18,19,20]. The role of these compounds in *T. pannonicum*’s adaptation and tolerance of salinity is not well investigated and the patterns of correlation between these compounds are not documented yet. There is a lack of knowledge of whether the activities of antioxidant enzymes are correlated with the concentration of salinity stress indicators during *T. pannonicum’s* adaptation to salinity. There is no evidence as to which organs (roots, stem, or leaves) are most affected by different NaCl concentrations. It is also not clear how the activity of antioxidant enzymes and salinity stress indicators (proline and H_2_O_2_) are correlated between organs and related to each other. The studies by Ievinsh et al. indicated a high dispersion of leaf water content, Na^+^ vs. K^+^ concentration in water from leaf tissue, and high sodium accumulation with low potassium levels in the leaves of the sea aster but without a dipper biochemical analysis that is crucial for understanding the salinity tolerance and adaptation [7]. 

Few studies have actually shown plant responses to different durations of salinization in short- and long-term periods. The researchers usually focus only on one type of response to salt stress, short- or long-term [21,22]. Therefore, in our study we want to explain how the short- and long-term NaCl stress act on the activity of antioxidant enzymes and on the concentration of salinity stress indicators. Examining the plant stress response on a wide time scale will allow us to fully understand the adaptation of *T. pannonicum* to extreme and variable soil salinity in the habitat. From the perspective of autecological studies on halophyte adaptation to salinity, the long-term stress effect seems to be even more significant than the short time salinity effect.

The main goal of our research was to determine at which levels (plant growth, organ, time) salinity can modify the stress response of *T. pannonicum*. We performed this autecological study to evaluate the following hypotheses: (1) even though *T. pannonicum* is a halophyte, salinity significantly affects germination and late growth, (2) salinity significantly affects the activity of antioxidant enzymes (APX, POD, and CAT) and salinity stress indicators (hydrogen peroxide and proline) in the root, stem and leaves of *T. pannonicum*, (3) the duration of salinity exposure modifies plant physiological responses. We hope that the results will provide a novel view to understanding the interactions of individual species with the extreme environment and to recognize the salinity tolerance of this plant.

## 2. Materials and Methods

### 2.1. Seed Collection

The seeds were collected in November 2019 from the anthropogenic inland saline habitat near a soda factory in the town of Inowrocław (52°48′ N, 18°15′ E). This site represents an industrial saline area in Poland, with salinity associated with waste from the soda production. In this area, the EC_e_ value reached even 140 dSm^−1^ (which corresponds to 1400 mM NaCl) [23]. Experiments were conducted at the Nicolaus Copernicus University in Torun, Poland in 2020. The permission to work with the seeds of a protected plant was provided by the regional director of the Environmental Protection in Bydgoszcz (WOP.6400.17.2020JC).

### 2.2. Germination Experiment

Prior to the germination experiment, cold stratification was performed (4 °C, 30 days). Then, the seeds were sown on Petri dishes (Ø 7 cm) containing Whatman No. 2 filter paper (three replicates of 35 seeds in each salinity variant). We watered the seeds with four variants of a solution: control (0 mM NaCl), 200, 400, and 800 mM NaCl. NaCl concentrations were selected according to those observed in the field studies [23]. Piernik et al. [23] indicated the minimum (ca. 2 dS/m), maximum (ca. 100 dS/m), and optimum (ca. 30 dS/m) salinity for *T. pannonicum* growth. These correspond, respectively, to 20, 1000, and 300 mM NaCl. However, we did not obtain seedlings in the germination experiment at 1000 mM NaCl. Therefore, we established finally an upper limit of 800 mM NaCl. The Petri dishes with seeds were put into the growth chamber with day/night (25 °C), a humidity of 50–60%, a photon flux density of 1000 mmol m ^−2^ s^−1^, and a photoperiod of 16/8 h (light/dark) (LED lights with white, full-spectrum light) [24]. The number of germinated seeds was determined daily (in the same part of the day) until the end of the 14th day after sowing.

The germination parameters were calculated based on the International Seed Testing Association (ISTA) method [25].

Germination percentage (GP):

GP = (n/N) × 100,(1)

where n is the number of normally germinated seeds and N is the number of all seeds sown.

Germination index (GI):

GI = ∑(G_t_/T_t_), (2)

where G_t_ is the number of seeds germinated on day t, and T_t_ is the number of days. 

Mean germination time (MGT):

MGT = ∑(T_i_ × N_i_)/∑N_i_, (3)

where N_i_ is the number of newly germinated seeds at time T_i_.

Germination energy (GE) was assessed on the fourth day by counting the number of typical seedlings according to the ISTA (2006) standard [25].

### 2.3. Pot Experiments

Following 14 days of germination, the seeds were transferred into individual pots (height: 5.3 cm, diameter: 5.5 cm) with a mixture of vermiculite and sand (1:1) as a substrate. Each pot was saturated to full capacity by solutions of 0, 200, 400, and 800 mM NaCl (ca 35 mL of solution for 1 pot with the substrate) to reflect the salinity of the soil in the field. For individual variants of salinity, we prepared six pots (total 6 pots × 4 variants of NaCl concentration). The pots were located on individual trays filled with 210 mL NaCl solution (35 mL × 6 pots). Because in the first step we saturated the pots to the full capacity with NaCl solution, the NaCl was still present in the “soil solution” and the concentration of NaCl in this medium did not change during the experiment [24,26,27]. Seedlings were irrigated for three months with 210 mL of Hoagland’s solution added to each tray every two days [22]. Following three months of plant development, the growth parameters and biochemical parameters were estimated per triplicate for each NaCl concentration (plants were randomly selected). We evaluated the salinity effect on six growth parameters: shoot length (SL), root length (RL), total fresh mass (FM_T_), shoot fresh mass (FM_S_), root fresh mass (FM_R_), and number of leaves in the rosette (No.L_R_); and on biochemical parameters: the activity of antioxidant enzymes (APX and POD) and salinity stress indicators (hydrogen peroxide and proline) in the roots, stems and leaves. 

The phenotype photos after treatment with different salt concentrations were performed with a Sony digital camera and processed according to Cárdenas-Pérez et al. [24].

### 2.4. Long- and Short-Term Effects of Salinity

The three-month-old plants (growing without NaCl application) were stressed with 800 mM NaCl and the leaves were harvested after 1 h, 3 h, and 5 h (short-term salinity stress), and after 24 h, 48 h, 5 days, and 7 days (long-term salt stress) of NaCl addition. Then, the antioxidant enzyme activities (POD and APX) and salinity stress indicators (hydrogen peroxide and proline) were assessed.

### 2.5. Biochemical Analysis

For the determination of the activity of antioxidant enzymes (POD and APX), the leaves, roots, and stems were homogenized in 50 mM potassium phosphate buffer pH 7.0, including 0.1 mM EDTA on ice in a mortar. Then, the homogenate was centrifuged at 15,000× *g* for 15 min at 4 °C. The obtained supernatant was used for the determination of the antioxidant enzyme activities and protein content. The peroxidase activity (POD) was examined according to Maehly and Chance [28]. The enzymatic reaction was initiated by adding 100 µL of supernatant to the mixture of 50 mM potassium phosphate buffer (pH 7.0), 20 mM guaiacol, and 40 mM H_2_O_2_. Changes in the absorbance of the reaction solution at 470 nm were read every minute. One unit of the enzyme activity was defined as the amount of enzyme causing a 0.001 change in absorbance per minute. The enzyme activity was presented as U·mg^−1^. The protein concentration was determined by the Bradford method [29]. The absorbance of the protein solution was measured at 595 nm with bovine serum albumin (BSA) as a standard. The ascorbate peroxidase activity (APX) was followed by the method of [30]. The assay mixture contained 0.1 mL of supernatant with enzyme, 0.1 mM EDTA, 0.5 mM ascorbate, 0.1 mM H_2_O_2_, and 1 mL of potassium phosphate buffer (pH 7.0). The decrease in the absorbance of ascorbate at 290 nm was measured and one unit of the enzyme activity was defined as the amount of enzyme causing a 0.001 change in absorbance per minute. The activity of APX was expressed as U·mg^−1^.

Hydrogen peroxide in plant organs was examined according to Sergiev et al. [31] with modifications described by Velikova et al. [32]. Plant tissues (500 mg) were homogenized in an ice bath with 5 mL 0.1% TCA. Then, the homogenate was centrifuged at 12,000× *g* for 15 min (4 °C), and 0.5 mL of the supernatant was added to 0.5 mL 10 mM potassium phosphate buffer (pH 7.0) and 1 mL 1 M KI. The solution was incubated in the dark for one hour and the absorbance of the supernatant was read at 390 nm. The final H_2_O_2_ concentration was expressed as µM.

The proline level was assessed according to the methodology of Abrahám et al. [33] with a small modification. Fresh plant material (500 mg) was homogenized on ice in a mortar with 3% aqueous sulfosalicylic acid solution (5 μL of solution per one mg of plant material). The homogenate was centrifuged (18,000× *g*, 10 min, 4 °C), and the supernatant was collected. The reaction mixture was composed of 2 mL of glacial acetic acid, 2 mL of acidic ninhydrin reagent, and 2 mL of supernatant. An acidic ninhydrin reagent was prepared according to Bates et al. [34]. The reaction mixture was shaken and incubated at 100 °C for 30 min. The reaction was inhibited by placing the samples on ice. To extract the chromophore, 4 mL of toluene was added and quantified spectrophotometrically at 520 nm. Proline concentrations were presented in µg/mL.

### 2.6. Detection of Catalase Activity by Non-Denaturing Polyacrylamide Gel Electrophoresis (PAGE) in Roots, Shoots, and Leaves

Leaves, roots, and stems were homogenized in 50 mM potassium phosphate buffer pH 7.0, including 0.1 mM EDTA on ice in a mortar. Then, the homogenate was centrifuged at 15,000× *g* for 15 min at 4 °C, and 10 µg of protein per sample was loaded on 6% resolving gel solution [35]. The electrophoresis was run at 15 mA at 4 °C for 2 h. Following electrophoresis, the gel was soaked in distilled water for 5 min at room temperature. Then, the gel was incubated with 100 mL of a solution with 4 mM H_2_O_2_ (10 min, RT) and washed with 100 mL of distilled water. Following this step, the gel was moved to 100 mL of a solution (1% (*w*/*v*) ferric chloride and 1% (*w*/*v*) potassium ferricyanide). When the gel turned dark green, the ferric chloride/potassium ferricyanide solution was removed and washed with distilled water. Bands of catalase activity were marked as clear bands and their intensity corresponded with the activity of CAT [36]. 

### 2.7. Statistical Analysis

One-way ANOVA with Tukey’s post hoc comparison was applied to estimate the significance of the differences in (1) germination and growth parameters, (2) the activity of antioxidant enzymes and concentrations of proline in the roots, stem, and leaves, and (3) the salinity stress duration. A principal component analysis (PCA) was used to determine the correlation pattern between traits, and then Pearson’s correlation coefficients were used for the correlation assessment. The statistical significance between treatments was agreed at the *p* < 0.05 level. For calculations, Statistica version 8.0 [37] and Canoco 5.0 [38] packages were used.

## 3. Results

### 3.1. Effect of Salinity on Two Growth Stages

Salinity affected similarly on the germination and late-growth parameters of *T. pannonicum*. The differences between treatments were significant in all measured parameters except FM_R_ (Table 1 and Table S1). Salinity significantly reduced GP, GI, and GE and increased MGT. Germination was highest at 0 mM NaCl, and lowest at 800 mM NaCl (Table 1). The MGT was highest at 800 mM NaCl, but in the control, 200 mM, and 400 mM NaCl, we observed no statistically significant differences. Salinity also significantly reduced the parameters of late growth: SL, RL, FM_T,_ and FM_S_. The stem was longest in the control (31.3 cm), and the roots were longest in the control and 200 mM NaCl (ca 8.3 cm). FM_T_ was highest in the control and 200 mM NaCl (ca 24 g) and lowest at 400 and 800 mM NaCl (ca 20 g). We observed no significant difference in FM_S_ and No.L_R_ between the control and 200 mM and between 400 mM and 800 mM NaCl (Table 1). FM_S_ was lowest at 400 and 800 mM NaCl (18 g and 16.7 g, respectively). FM_R_ was not significantly affected by salinity. 

### 3.2. Effect of Salinity on Organ Stress Responses

**Figure 1:**
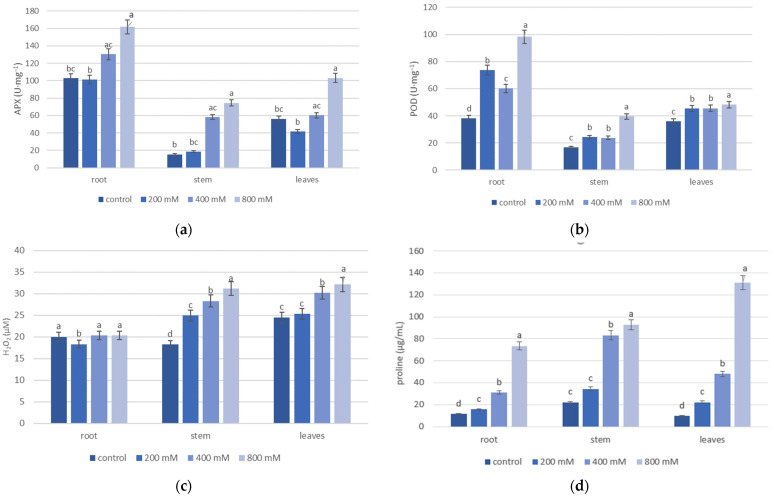
Effect of NaCl on the activity of antioxidant enzymes—(**a**) APX and (**b**) POD, and salinity stress indicators—(**c**) H_2_O_2_ and (**d**) proline in the roots, stems, and leaves of *T. pannonicum*. Differences between groups based on Tukey’s range test are marked by different letters and are significantly different at *p* ≤ 0.05. One unit of the enzyme activity was defined as the amount of enzyme causing a 0.001 change in absorbance per minute. Average values with standard error are given *n* = 3.

**Figure 2:**
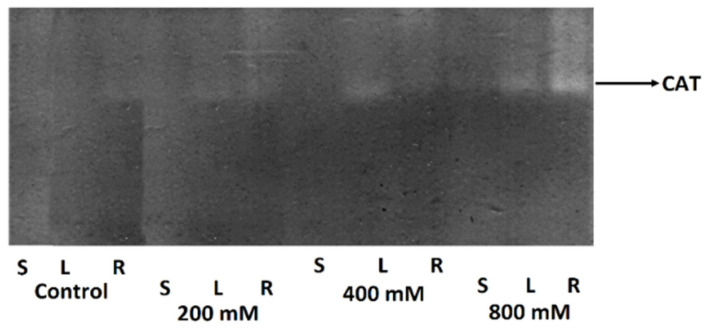
Zymogram of CAT enzyme activity in the roots (R), stem (S), and leaves (L) after 0 (control), 200, 400, and 800 mM NaCl treatment. Bands of catalase activity were marked as clear bands and their intensity corresponded with the activity of CAT (shown by the arrow) between organs.

Salinity significantly affected the activity of antioxidant enzymes (APX and POD) and concentrations of salinity stress indicators (hydrogen peroxide and proline) in the roots, stems, and leaves of *T. pannonicum* (Table S2). The main effects of salinity on the organs, and the interaction effects between them, were significant for all measured parameters (Table 2). In all organs, salinity increased APX and POD activity. The activity of APX and POD was highest at 800 mM NaCl in all analyzed plant organs (Figure 1). In addition, the zymogram analysis indicated that CAT enzyme activity was also highest at 800 mM NaCl (Figure 2).

Of all organs, the activity of APX and POD was lowest for the stem in the control (Figure 1a,b). The lowest activity of APX in the root and leaves was observed at 200 mM NaCl. The activity of POD for all organs was lowest in the control. We observed no significant difference in POD activity in the leaves in 200 and 400 mM NaCl (Figure 1b). The highest intensity of the bands in the CAT zymogram was observed for the roots after the 800 mM NaCl treatment. In addition, we detected one isoform of catalase in all NaCl treatments (Figure 2). The highest concentrations of H_2_O_2_ and proline were observed at 800 mM NaCl in all analyzed organs (Figure 1c,d). The lowest concentration of H_2_O_2_ between all organs was noticed for the stem in the control (18.2 µM). The lowest H_2_O_2_ concentration in the root and leaves was observed in 200 mM NaCl (Figure 1c). The concentration of H_2_O_2_ in the root seems to be independent of the increasing salinity because no significant difference was observed between NaCl treatments. The amount of proline for all organs was lowest in the control (11.4 µg/mL for the root, 21.8 µg/mL for the stem, and 9.49 µg/mL for the leaves). For all organs, proline concentration increased with the growing salinity and was greatest in the leaves (Figure 1d).

### 3.3. Effect of the Duration of Salinity on the T. pannonicum Stress Responses

Based on the above result of our experiment, we investigated the effect of long- and short-term salt exposure on the enzyme activity, and the concentration of hydrogen peroxide and proline in the leaves as the main organ affected by salinity and the organ responsible for salt extrusion by the shedding of rosette old leaves saturated by salt [39]. Because the most significant effect of the salinity was obtained for 800 mM NaCl, we selected this concentration of salt for further analysis. 

**Figure 3:**
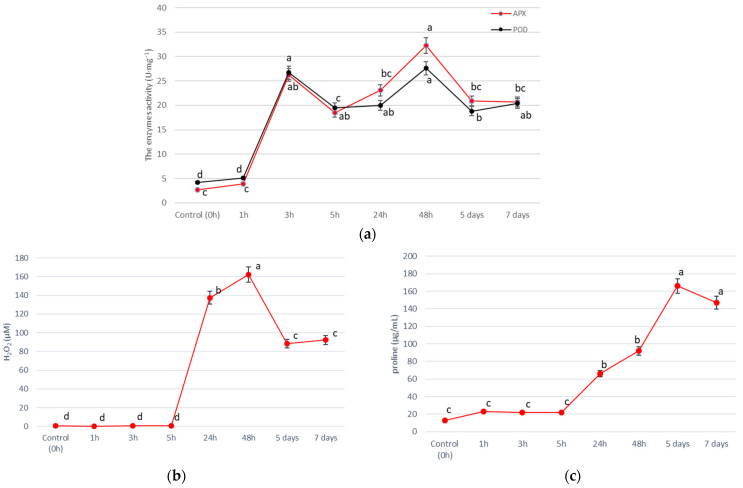
Effect of the duration of salinity exposure on the activity of antioxidant enzymes—(**a**) APX and POD, and salinity stress indicators—(**b**) H_2_O_2_, and (**c**) proline, in the leaves of *T. pannonicum*. Differences between groups based on Tukey’s range test are marked by different letters and are significantly different at *p* ≤ 0.05. One unit of the enzyme activity was defined as the amount of enzyme causing a 0.001 change in absorbance per minute. Average values with standard error are given *n* = 3.

The duration of salinity exposure significantly affected the activity of antioxidant enzymes and salinity stress indicator concentrations (Table S3). We observed that salinity acts on analyzed parameters over different time scales (Figure 3). The activities of both APX and POD enzymes were similar for each analyzed timeframe, however, significant differences in the APX activity, between 5 h and 48 h, were observed (Figure 3a). The highest activity of APX and POD was observed 1 h after NaCl application (short-term salt stress) (26.2 U·mg^−1^ for APX and 26.7 U·mg^−1^ for POD) and 48 h (long-term salt stress) (32.3 U·mg^−1^ for APX and 27.6 U·mg^−1^ for POD). The highest concentrations of H_2_O_2_ and proline were at 48 h and in 5 days after NaCl application, respectively (Figure 3b,c). There were no significant differences in the salinity stress indicator concentrations in short-term salt stress (1 h, 3 h, and 5 h), nor in long-term salt stress (5 days and 7 days). 

### 3.4. Patterns of Correlation between Growth Stage, Organs, and Duration of Salinity Exposure

**Figure 4:**
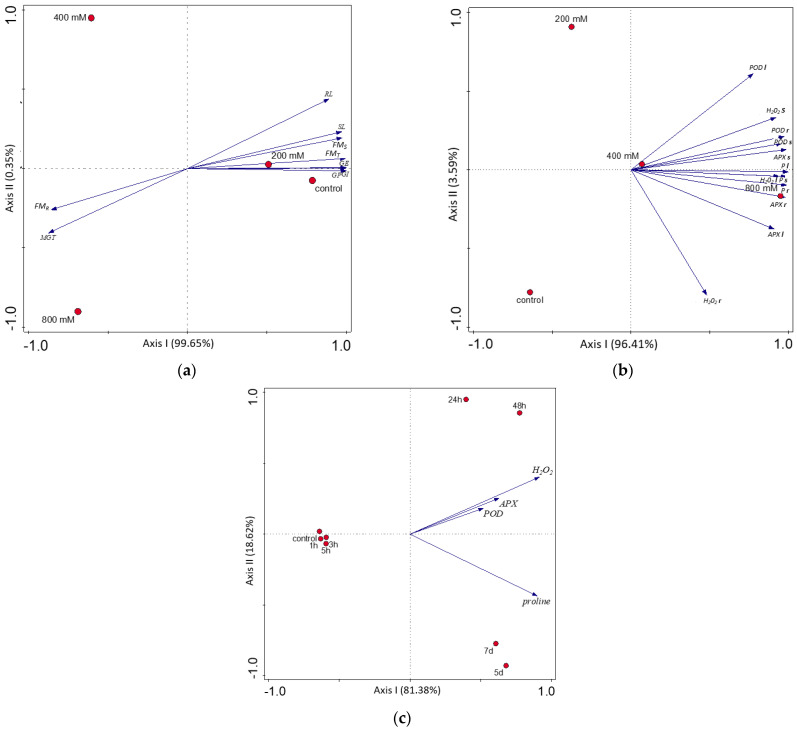
Result of the PCA analysis between: (**a**) salinity treatments and germination and growth parameters; (**b**) salinity treatments and stress responses in different organs; (**c**) duration of salinity exposure and stress responses in the leaves. Abbreviations as in Table 1 and Table S2. The concentrations of NaCl (**a**,**b**) and duration of NaCl stress (**c**) are marked by red points.

All of the variables (germination and growth parameters, antioxidant enzymes, and salinity stress indicators in organs) were evaluated in the NaCl concentration, while biochemical parameters were also evaluated in the time scale using PCA. The response to salinity was dependent on the growth stage, organs, and duration of salinity stress, where the first ordination axis represents the salinity gradient (Figure 4a–c). 

We noticed a strong and statistically significant positive correlation between POD in the roots and the concentration of H_2_O_2_ in the stem (Pearson’s r = 0.864) and a very strong positive correlation between APX in the roots and the concentration of H_2_O_2_ in the leaves (Pearson’s r = 0.961). The concentration of proline was also correlated significantly with the activity of antioxidant enzymes, especially with APX activity. A very strong positive correlation was observed for the proline concentration in the stems and APX activity in the roots (Pearson’s r = 0.990) and for the proline concentration in the leaves and APX activity in the roots (Pearson’s r = 0.985). The concentration of salinity stress indicators (H_2_O_2_ and proline) was positively correlated between organs. The strongest significant correlation was found for the concentration of proline in the stem and H_2_O_2_ in the leaves (Pearson’s r = 0.981) (Figure 4b, Table S5). The activity of antioxidant enzymes and concentration of salinity stress indicators were strongly correlated with the type of salinity response early up to 5 h and late from 24 h, represented by the first PCA axis explaining ca 81% of the traits’ variance (Figure 4c). The differentiation in the late response, represented by the second PCA axis, explained ca 18%. All analyzed parameters were positively correlated with the duration of salinity stress (Figure 4c, Table S6). The activities of APX and POD were significantly and very strongly correlated with each other (Pearson’s r = 0.984). The positive correlation was high between APX and H_2_O_2_ (Pearson’s r = 0.666), and moderate between POD and H_2_O_2_ (r = 0.532). The correlation between two salinity stress indicators (proline and H_2_O_2_) was also significant and moderate (r = 0.385; Table S6). 

## 4. Discussion

### 4.1. Salinity Affects Germination and Late Growth of T. pannonicum

Germination is a crucial part of the plants’ growth, but more so for halophytes, and salt tolerance usually varied between halophytes [40]. To survive in a saline habitat, halophytes required successful seed germination [41]. Most parameters of germination and growth were affected by salinity (Table 1 and Table S1). These results are in line with our previous studies on glycophytic species, e.g., fodder beet [42], sorghum [26], maize, millet, and oat [2]. In our study, the effective *T. pannonicum* seed germination was achieved in the control (0 mM NaCl) although the model plant is an obligatory halophyte. The negative effect of salinity was also visible in the reduction of the germination energy (Table 1), because reducing the osmotic potential of the solution inhibits the imbibition of water by the seeds. MGT parameter (determining the time for the seed to germinate) was almost the same in the control, 200 mM and 400 mM NaCl (Table 1) which can be an example of a *T. pannonicum*’s seed strategy for efficient germination even under high soil salinity. Salinity had a significant effect on germination time (Table 1), as in a study of *P. sativum* and *L. sativus* [14]. The higher NaCl concentrations lengthen the germination time until the seeds develop a tolerance and start to germinate. Faster and early germination under lower salinity confers an ecological advantage upon halophyte seedlings [40].

Our study has shown that salinity strongly reduces the growth parameters of three-month-old plants, and maximum growth is obtained under non-saline and low-saline conditions (Table 1) as in a study by Geissler et al., 2009 [43], not all plant growth parameters were affected by salinity at the same level. The best growth parameters were observed in the control and 200 mM NaCl, which indicates the optimal concentration of NaCl for growth success. In addition, the total biomass of *T. pannonicum* was similar in 400 mM and 800 mM NaCl, however lower than in the control and 200 mM NaCl, indicating effective adaptation to higher levels of salinity. High variability of salinity in the *T. pannonicum* wet habitats promotes a broad spectrum of NaCl tolerance which was observed also by Ievinsh et al. [7]. They found *T. pannonicum* also in habitats with low salinity [7]. In addition, Karlsons et al. 2008 [44] demonstrated a higher decrease in the roots and leaves biomass of this species at 400 mM NaCl, compared with our studies. However, the adaptations to environmental stress can evolve within populations of the same species [45] and can be genetically established within a population as the result of local adaptation. 

### 4.2. Response to Salinity Depends on the Organs of T. pannonicum

The result of our study demonstrated that the halophyte’s organs do not simply tolerate high-salt conditions (Table 2 and Table S2). All biochemical parameters (activity of APX and POD, concentration of H_2_O_2_ and proline) in the analyzed organs were affected by salinity. The higher salt tolerance of numerous halophytes is related to proper ROS homeostasis by the activation of their antioxidant systems under salt stress [22]. The highest activities of APX, POD, and CAT were observed at 800 mM NaCl in all analyzed plant organs and were greatest for the roots (Figure 1a,b and Figure 2). The activities of APX and POD observed at 400 mM and 800 mM NaCl were varied between organs and revealed the following activity pattern: root > leaves > stem (Figure 1). Increased antioxidant activity has been observed in several salt-tolerant plants, indicating that antioxidants are an important factor in the salt stress response [46]. Enzymatic oxidative stress defenses to high salt concentration are more generally in the roots (such as CAT or APX activation), while root-specific antioxidant enzymes (i.e., SOD, and MDHAR) have also been found [47]. 

As shown in our study, the highest concentration of salinity stress indicators (H_2_O_2_ and proline) was observed at 800 mM NaCl in all analyzed organs (Figure 1c,d). Salt stress also generated oxidative stress in halophytes of the genus *Juncus* [48]. The highest efficiency of the antioxidant system in the root was the reduced H_2_O_2_ concentration in this organ which indicated that ROS scavenging mechanism is most effective in the roots, compared to the stems and leaves. An interesting observation is that proline was preferentially accumulated in the leaves (Figure 1d), however, in increasing NaCl concentrations, we noticed proline accumulation in all organs. Our results indicated that, in the context of proline accumulation, leaves are more salt-tolerant than other organs. Organ-specific accumulation of proline in the leaves of lupine and halophyte *K. virginica* was also observed by Rady et al., 2016 [18] and Wang et al., 2015 [49] and H_2_O_2_ in the leaves of rice [50]. 

### 4.3. Response to Salinity Depends on the Duration of Salinity Stress

Studies of the effects of salinity on halophytes on a wide time scale (in short-time and long timescale) are significant to fully understand the adaptation strategy and plant interaction with the environment. The response of *T. pannonicum* to salinity stress was two stages to enable more efficient and comprehensive adaptiveness (Figure 3a–c). The H_2_O_2_ concentration peaked at 48 h (long-term salt stress), which corresponds with the increased activity of APX and POD, the key enzymes responsible for H_2_O_2_ scavenging during salinity stress in plants. A significant result is that the activity of both investigated peroxidases is synergistic (Figure 3a). It seems reasonable to cope with NaCl stress “with redoubled strength”. It is also evidence of an early- and late-cellular stress response, which was for the first time demonstrated for *T. pannonicum*. Similar onset/reinforcement of antioxidant systems were also observed for groundnut [12] and soybean [51] under salt stress. For halophyte *S. aralocaspica*, adaptation to the external salinity changes for a period of 24–48 h has been reported [52]. Hernández et al., 2000 [53] noticed that the induction of antioxidant defence is at least one component of the tolerance mechanism of *Pisum sativum* L. to long-term salt stress. According to Fraire-Velazquez and Emmanuel [17], the observed initial, fast response to salinity stress is temporary and thus separated clearly from the pathological consequences of exposure to the same level of salinity over a longer period, which is catastrophic for non-adapted plants. 

The highest concentrations of H_2_O_2_ (162.1 µM) and proline (166.08 µg/mL) were recorded at 48 h and 5 days after salinity treatment, respectively. Huang et al., 2013 [20] observed the same dependencies in *H. tuberosus* during the initial 72-h period. Compared with enzyme activity, the increase in the concentration of salinity stress indicators was a part of the late stress response (Figure 3b,c) therefore these compounds may be more responsible for the long-term adaptation process of the plant to the extreme environment than antioxidant enzymes, as in the study of Naliwajski and Skłodowska 2021 [54]. The action of H_2_O_2_ and proline is also synergistic to intensify the defense mechanisms against NaCl stress. Studies by Huang et al. 2013 [20] demonstrated that the gene expression of key enzymes in the proline biosynthetic pathway changed significantly in roots of *H. tuberosus* after 4 h treatment, which may be responsible for the increase in the concentration of proline observed after 5 h of salinity treatment in our study (Figure 3b).

### 4.4. Salt-Tolerance and Salt-Adaptation of T. pannonicum

We indicated that germination success determines the future plant biomass (FM_T_) and biomass of shoots (FM_S_) (Table S4). Bayuelo-Jiménez et al., 2002 [15] observed that faster germination allowed emerging seedlings to obtain a higher biomass. The strong negative correlation observed in our study for RL and MGT (Table S4) corresponds with the findings by Robin et al., 2016 [55]. Salinity induced a reduction in the surface area of the root and changes in the main root in wheat [55], which may explain the extended mean germination time of *T. pannonicum* seeds under salinity. Although *T. pannonicum* is a halophyte, saline conditions at the germination stage will be defined as the further adaptation success of this plant. Low soil salinity under the first step of plant growth allowed this plant to obtain a better biomass and overcome interspecific competition in the environment. A PCA analysis indicated a correlation between the activity of antioxidant enzymes and proline concentrations in the investigated organs under NaCl stress, which can be essential for *T. pannonicum* adaptation (Figure 4b, Table S5). The early cellular stress response observed after 1 h and 5 h of NaCl exposure (Figure 3a–c and Figure 4c) can help the plant to restore its performance more quickly and is essential to stress neutralization and further plant survival [17]. Then, the changes in enzyme activities affecting the concentrations of salinity stress indicators (H_2_O_2_ and proline) were part of the late cellular stress response observed 24 h after salinity stress (Figure 3a–c). The first line of defense against ROS caused by oxidative stress comprises antioxidant enzymes, so they are activated more quickly than the production/accumulation of proline [56]. The growth response to salinity stress can be divided into two steps: a fast reaction to the increase of the external osmotic pressure, and a slower response as a result of the accumulation of Na^+^ in plant organs, which was documented by Munns and Tester 2008 [57]. The results of our studies strongly indicate that the levels of stress indicators and the activity of antioxidant enzymes contribute to the tolerance and adaptation of *T. pannonicum* to salinity stress. 

The high salt tolerance was noticed for the germination and late growth of *T. pannonicum* results from its natural growth in very stressful and variable habitats. *T. pannonicum* is part of inland and coastal plant communities [8,9,23]. The ecological habitats of sea aster are extremely unfavorable: inland saline meadows flooded in the spring after snowmelt and dried in the summer, or coastal seashores with sea tides. This species in anthropogenic areas can be also exposed to salty waste from the soda or potassium industry [58]. These difficult growth conditions increase the adaptability of *T. pannonicum.* Investigated salt-adaptation traits, such as a high total biomass under salinity, the high and organ-specific activities of APX, POD, and CAT, and the highest efficiency of the antioxidant system in the root with a leaves-specific accumulation of proline, are examples of plant answers for the high variability of the habitats where they grow. 

## 5. Conclusions

*T. pannonicum* is a member of a diverse group of halophytes that seems to be a promising cash crop to desalinize and reclaim degraded land. However, some basic physiological, biochemical, and molecular mechanisms in the adaptive processes of *T. pannonicum,* such as salt tolerance, are still not well recognized. Our study and recent genetic and omic experiments have indicated that salt tolerance is a complex mechanism that depends on the growth phase, organs, and duration of salinity exposure. The antioxidant system of *T. pannonicum* was very active at 800 mM NaCl and APX. POD, and CAT activity were greatest for the roots. The demonstrated different responses of the organs to NaCl application have significant potential to further proteomic and metabolomic analyses of halophyte adaptions. The time-dependent regulation of adaptive processes involved in the tolerance of high and extended salinity, but also shorter episodes of salinity, also requires a deeper explanation. A better understanding of the adaptabilities of *T. pannonicum* to high salinity could be helpful in the restoration of the fragmented aster population and studies of these plants’ application as energy crops for cultivation on saline lands or as cash crop vegetables. 

## Figures and Tables

**Table 1:** Effect of NaCl on the germination and growth parameters of *T. pannonicum*.

Trait	Control	200 mM	400 mM	800 mM	ANOVA
			NaCl		
Germination parameters					
GP	61.7 ^a^ ± 0.88	50.3 ^b^ ± 0.33	3.67 ^c^ ± 0.33	1.33 ^d^ ± 0.67	*p* < 0.0001
GI	6.65 ^a^ ± 0.18	5.85 ^b^ ± 0.03	0.42 ^c^ ± 0.056	0.053 ^d^ ± 0.01	*p* < 0.0001
MGT	4.89 ^b^ ± 0.045	4.49 ^b^ ± 0.04	5.66 ^b^ ± 0.29	7.33 ^a^ ± 0.17	*p* < 0.0001
Plant morphology	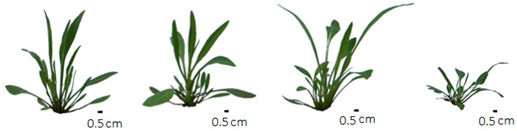				
Growth parameters					
SL (cm)RL (cm)	31.3 ^a^ ± 0.67	28.5 ^b^ ± 0.29	21.0 ^c^ ± 0.58	16.3 ^d^ ± 0.21	*p* < 0.0001
	8.27 ^a^ ± 0.15	8.30 ^a^ ± 0.12	6.83 ^b^ ± 0.18	5.07 ^c^ ± 0.09	*p* < 0.0001
FM_T_ (g)	24.4 ^a^ ± 0.64	23.4 ^a^ ± 0.37	20.9 ^b^ ± 0.26	20.04 ^b^ ± 0.13	*p* = 0.0003
FM_S_ (g)	21.3 ^a^ ± 0.50	21.1 ^a^ ± 0.43	18 ^b^ ± 0.35	16.7 ^b^ ± 0.35	*p* = 0.0002
FM_R_ (g)	2.83 ^ab^ ± 0.43	2.40 ^b^ ± 0.12	3.29 ^ab^ ± 0.30	3.66 ^a^ ± 0.23	*p* = 0.1077
No.L_R_	18 ^a^	17 ^a^	16 ^b^	15 ^b^	*p* < 0.0001

Average values (*n* = 3) with standard error (SE) are given. GP = germination percentage; GI = germination index; MGT = mean germination time; GE = germination energy; SL = shoot length; RL = root length; FM_T_ = total fresh mass; FM_S_ = shoot fresh mass; FM_R_ = root fresh mass; No.L_R_ = number of leaves in a rosette. Differences between groups based on Tukey’s range test are marked by different letters and are significantly different at *p* ≤ 0.05.

**Table 2:** Analysis of variance (mean squares) for the activity of antioxidant enzymes and salinity stress indicators in two organs (O) of *T. pannonicum* and four salt concentrations (S).

Trait	Sources of Variations			
	S	O	SxO	Error
df	2	4	8	24
APX	10,691 **	11,643 **	1824 **	1.082
POD	15.44 **	52.15 **	3.56 **	0.096
H_2_O_2_	97.73 **	221.34 **	26.14 **	0.409
P	13,145.01 **	3869.41 **	437.55 **	31.615

Average values (*n* = 3) with errors within group variance are given. APX = ascorbate peroxidase activity; POD = peroxidase activity; H_2_O_2_ = H_2_O_2_ concentration; P = proline concentration; df = degrees of freedom; ** = *p* ≤ 0.01.

## Data Availability

The datasets generated during the current study are available from the corresponding author upon reasonable request.

## Supplementary Materials

The following are available online at https://www.mdpi.com/article/10.3390/life13020462/s1, Table S1: Analysis of variance (mean squares) for the germination and the growth parameters of *T. pannonicum* in four salt concentrations, Table S2: Analysis of variance (mean squares) for the activity of antioxidant enzymes (APX and POD) and salinity stress indicators (H_2_O_2_ and proline) of *T. pannonicum* in four salt concentrations, Table S3: Analysis of variance (mean squares) for the activity of antioxidant enzymes (APX and POD) and salinity stress indicators (H_2_O_2_ and proline) of *T. pannonicum* in eight different times of measurements, Table S4: Correlation coefficient matrix between germination and growth parameters under NaCl stress, Table S5: Correlation coefficient matrix between antioxidant enzymes and salinity stress indicators under NaCl stress, Table S6: Correlation coefficient matrix between antioxidant enzymes and salinity stress indicators under the different times of the duration of salinity stress.
