# Supplementary material for: Growth Stage-, Organ- and Time-Dependent Salt Tolerance of Halophyte Tripolium pannonicum (Jacq.) Dobrocz
Source: Life (Basel). 2023 Feb 7;13(2):462. doi: 10.3390/life13020462 (PMC9962771; doi:10.3390/life13020462)
Supplement: Supplementary file 1 [file life-13-00462-s001.zip › life-2163750-supplementary.pdf]

Table S1. Analysis of variances (mean squares) for the germination and the growth parameters of *T. pannonicum* in four salt concentrations.

Trait		Sources of Variations S	Error
	df	3	8
Germination parameters	GP	2929 **	1.08
	GI	36.5 **	0.030
	MGT	17.4 **	0.252
	GE	346 **	0.167
Growth parameters	SL	141 **	0.729
	RL	7.00 **	0.065
	FM_T	11.5 **	0.492
	FM_S	15.8 **	0.649
	FM_R	0.910 n.s	0.323

GP= Germination percentage; GI= Germination index; MGT= Mean germination time; GE= Germination energy; SL= Shoot length; RL= Root length; FM_T = Total Fresh mass; FM_S = Shoot fresh mass; FM_R = Root fresh mass; S = salinity; df = degrees of freedom; ns = non-significant; Error = within group variance. ** = $p \leq 0.01$.

Table S2. Analysis of variances (mean squares) for the activity of antioxidant enzymes (APX and POD) and salinity stress indicators (H_2O_2 and proline) of *T. pannonicum* in four salt concentrations.

Trait	Sources of Variations S	Error
df	3	8
APX_R	2424 **	0.707
APX_S	9830 **	0.758
APX_L	2086 **	1.78
POD_R	18.9 **	0.228
POD_S	2.78 **	0.031
POD_L	0.89 **	0.027
H_2O_{2R}	2.76 *	0.569
H_2O_{2S}	106 **	0.434
H_2O_{2L}	41.1 **	0.224
P_R	2396 **	0.678
P_S	7554 **	86.2
P_L	4069 **	7.9

APX_R = Root ascorbate peroxidase enzyme activity; APX_S = Steam ascorbate peroxidase enzyme activity; APX_L = Leaf ascorbate peroxidase enzyme activity; POD_R = Root peroxidase enzyme activity; POD_S = Shoot peroxidase enzyme activity; POD_L = Leaf peroxidase enzyme activity; H_2O_{2R} = Root H_2O_2 concentration; H_2O_{2S} = Steam H_2O_2 concentration; H_2O_{2L} = Leaf H_2O_2 concentration; P_R = Root proline concentration; P_S = Steam proline concentration; P_L = Leaf proline concentration; S = salinity; df = degrees of freedom; ns = non-significant; Error = within group variance. * = $p \leq 0.05$, ** = $p \leq 0.01$.

Table S3. Analysis of variances (mean squares) for the activity of antioxidant enzymes (APX and POD) and salinity stress indicators (H_2O_2 and proline) of *T. pannonicum* in eight different times of measurements.

Trait	Sources of Variations	
	Time	Error
df	7	56
APX	3.21 **	0.182
POD	2.31 **	0.238
H_2O_2	369 **	3.59
P	29448 **	833

APX= Ascorbate peroxidase enzyme activity; POD= Peroxidase activity; H_2O_2= H_2O_2 concentration; P= proline concentration; Time = eight different times of measurements; df = degrees of freedom; Error = within group variance, ** = $p \leq 0.01$.

Table S4. Correlation coefficients matrix between germination and growth parameters under NaCl stress.

Pearson's r	GP	GI	MGT	GE	SL	RL	FM_T	FM_S	FM_R
GP	-	* 0,998	-0,870	* 1,000	* 0,968	0,885	* 0,995	* 0,971	-0,856
GI	0,998	-	-0,889	* 0,998	* 0,969	0,897	* 0,991	* 0,979	-0,883
MGT	-0,870	-0,889	-	-0,874	-0,935	* -0,989	-0,880	* -0,962	* 0,973
GE	1,000	0,998	-0,874	-	* 0,973	0,891	* 0,997	* 0,973	-0,855
SL	0,968	0,969	-0,935	0,973	-	* 0,963	* 0,985	* 0,987	-0,879
RL	0,885	0,897	-0,989	0,891	0,963	-	0,907	* 0,967	-0,932
FM_T	0,995	0,991	-0,880	0,997	0,985	0,907	-	* 0,975	-0,844
FM_S	0,971	0,979	-0,962	0,973	0,987	0,967	0,975	-	-0,939
FM_R	-0,856	-0,883	0,973	-0,855	-0,879	-0,932	-0,844	-0,939	-

Color in the matrix highlights the sign and magnitude of coefficients. Red indicates a negative correlation, blue indicates positive values. The intensity of the color represents the magnitude of the value when the darker is more extreme. Abbreviations as in Supplementary Table S1. Statistical significance was assessed at the $p < 0.05$ and marked by a star.

Table S5. Correlation coefficients matrix between antioxidant enzymes and salinity stress indicators under NaCl stress.

Pearson's r	APX root	APX stem	APX leaves	POD root	POD stem	POD leaves	H_20_2 root	H_20_2 stem	H_20_2leaves	proline root	proline stem	proline leaves
APX root	-	0,624	0,941	0,739	0,891	0,671	0,636	0,838	* 0,961	* 0,978	* 0,990	* 0,985
APX stem	0,624	-	0,817	* 0,644	* 0,751	0,253	0,225	0,323	0,393	* 0,750	* 0,560	* 0,643
APX leaves	0,941	0,817	-	0,661	0,850	0,452	0,655	0,635	0,814	* 0,955	0,891	0,909
POD root	0,739	0,644	0,661	-	0,956	0,895	-0,049	* 0,864	0,699	0,834	* 0,790	* 0,844
POD stem	0,891	0,751	0,850	0,956	-	0,815	0,231	0,866	0,821	* 0,957	0,909	* 0,951
POD leaves	0,671	0,253	0,452	0,895	0,815	-	-0,053	0,959	0,758	0,696	0,765	0,774
H_20_2 root	0,636	0,225	0,655	-0,049	0,231	-0,053	-	0,232	0,610	0,501	0,557	0,491
H_20_2 stem	0,838	0,323	0,635	0,864	0,866	0,959	0,232	-	0,912	0,826	0,906	0,898
H_20_2leaves	0,961	0,393	0,814	0,699	0,821	0,758	0,610	0,912	-	0,901	* 0,981	0,950
proline root	0,978	0,750	0,955	0,834	0,957	0,696	0,501	0,826	0,901	-	* 0,968	* 0,988
proline stem	0,990	0,560	0,891	0,790	0,909	0,765	0,557	0,906	0,981	0,968	-	* 0,993
proline leaves	0,985	0,643	0,909	0,844	0,951	0,774	0,491	0,898	0,950	0,988	0,993	-

Color in the matrix highlights the sign and magnitude of coefficients. Red indicates a negative correlation, blue indicates positive values. The intensity of the color represents the magnitude of the value when the darker is more extreme. Statistical significance was assessed at the $p < 0.05$ and marked by a star. APX= Ascorbate peroxidase enzyme activity; POD= Peroxidase activity, H_2O_2= H_2O_2 concentration.

Table S6. Correlation coefficients matrix between antioxidant enzymes and salinity stress indicators under the different times of the duration of salinity stress.

Pearson's r	APX	POD	H_2O_2	proline
APX	-	* 0,984	* 0,666	0,455
POD	0,984	-	*0,532	0,647
H_2O_2	0,666	0,532	-	*0,385
proline	0,455	0,647	* 0,385	-

Color in the matrix highlights the sign and magnitude of coefficients. Red indicates a negative correlation, blue indicates positive values. The intensity of the color represents the magnitude of the value when the darker is more extreme. Statistical significance was assessed at the $p < 0.05$ and marked by a star. APX= Ascorbate peroxidase enzyme activity; POD= Peroxidase activity, H_2O_2= H_2O_2 concentration.
